# Construction and Validation of the Intimate Images Diffusion Scale Among Adolescents

**DOI:** 10.3389/fpsyg.2019.01485

**Published:** 2019-06-28

**Authors:** María Penado, María Luisa Rodicio-García, María Marcos Cuesta, Tania Corrás

**Affiliations:** ^1^Facultad de Ciencias de la Salud, Universidad Isabel I, Burgos, Spain; ^2^Unidad de Investigación Formación y Orientación para la Vida, Departamento de Didácticas Específicas y Métodos de Investigación y Diagnóstico en Educación, Universidade da Coruña, A Coruña, Spain

**Keywords:** sexting, adolescents, sexual behavior, sexuality, technology, assessment, validation

## Abstract

The digital age has produced changes in the way of relating between adolescents, causing the appearance of problems such as cyberbullying, addiction to social networks or the diffusion of images of personal and intimate content without consent. The aim of this research is the estimation of this last phenomenon through the construction and validation of a scale that estimates the prevalence with which adolescents exchange images of sexual content through mobile phone, chats or social media (Instagram or Facebook, among others). Through a sample of 602 adolescents aged between 12 and 19 years (*M* = 14.92, *SD* = 1.591), an analysis of the reliability of the scale was performed using Cronbach Alpha statistic as well as a confirmatory factor analysis. The scale showed high reliability (α = 0.976; Ω = 0.981) and makes an estimation of the prevalence of images exchange through 20 items that, for the first time, identify the relationship with the person who publishes or disseminates the images and the person in it. Results also showed that the diffusion and exchange of images are more frequent than the exchange of text messages with sexual content. In addition, differences had been found in terms of gender, since girls publish and send intimate images more frequently while boys are the ones that most frequently receive this type of content. In short, a scale with good psychometric properties had been developed to estimate the use of social networks and mobile phone for the diffusion of images of sexual content in the adolescent population.

## Introduction

The way in which adolescents relate with each other has undergone important transformations, mainly due to the use of mobile phones and digital devices as a way of communication. Despite the advantages and multiple possibilities of digital communication, its inappropriate use can cause serious problems in a population that is particularly sensitive and still in process of social learning (Walsh et al., 2008; Ortega-Ruiz and Núñez, 2012; Gámez-Guadix et al., 2013; Rothman et al., 2015).

One of the multiple phenomena that can occur among adolescents through digital media and that has aroused the interest of the scientific community, becoming an object of study in recent years, has to do with sending and receiving messages of explicit sexual content, phenomenon baptized as sexting (Garner, 2011). Although the original definition greatly restricts the type of phenomenon that can be understood as sexting (sending erotic or sexual messages or photographs), the authors currently maintain that the phenomenon of sexting has a broader perspective that includes sending, receiving and re-sending erotic or sexual content (including photographs, videos or messages) to other people through the use of mobile devices, tablets, social networks or other electronic media (Lenhart, 2009; Weisskirch and Delevi, 2011; Fleschler-Peskin et al., 2013), it is precisely this broad definition that will be use in this work.

The variability in the definition of sexting (Lounsbury et al., 2011) makes its estimation of the prevalence very difficult, with studies that have measured sending messages versus sending photos (Henderson and Morgan, 2011; Drouin and Landgraff, 2012), or others with an absence of the description of the person to whom they are sent or from whom the messages are received (ignorance of the age, totally alien person, partners or ex-partners, or the fact of being a man or a woman) (Klettke et al., 2014).

The prevalence observed in studies with adolescents who interact with messages of sexual content or suggestive photos ranges between 10 and 40% depending on whether the estimated behavior is sending or receiving photographs, as well as depending on the age of the participants. In this way, Kopecký (2011), from a sample of 9353 participants between 11 and 17 years who were students of primary and secondary schools, observed one of the lowest prevalence in the phenomenon of sending photos (9.7%). In the study carried out by Dake et al. (2012), with a sample of 1329 adolescents between 12 and 18 years, the prevalence of sending and receiving photos was increased to 17% of the sample surveyed. Temple et al. (2012) placed in 27.6% the percentage of subjects between 14 and 19 years old who have sent photos of sexual content.

Finally, it should be noted that the highest prevalence observed in the studies so far is the one obtained by Rice et al. (2012) through a sample of 1839 students between 14 and 17 years old and where it is observed that sending photos is around 17.8% while the reception of images of intimate content amounts to 41% of the participants. In the meta-analysis conducted by Handschuh et al. (2019) the results show different prevalences based on type of sexting. The prevalence of receiving photos or videos of sexual content is 44%, twice the prevalence for the sending of the same sexual content (22%). When young adults are included in the sample, the results show that the 82.23% of the participants reported having sexted at least once (Morelli et al., 2016). The differences in prevalence observed are justified due to the possible differences between the reception of images of intimate and personal content (*Passive sexting*) or the sending and dissemination of images of personal and intimate content (*Active Sexting*). In this sense, there are numerous studies that support a higher frequency of passive than active sexting with percentages that range between 30 and 40% for the first case and around 20% for active sexting. Behaviors such as the re-sending or diffusion of images that are not their own, and that therefore would not be considered as sexting active, are the most minority of those previously considered, with percentages that do not exceed 10% of the sample surveyed (Fleschler-Peskin et al., 2013; Harris et al., 2013; Temple and Choi, 2014; Villacampa and Gómez, 2016).

Attending to the identity of the individuals with whom the exchange of messages with sexual content takes place, the adolescents say they carry out the exchange mainly with their partners or people with whom they have a romantic relationship (Cox-Communications, 2009; Fleschler-Peskin et al., 2013; Harris et al., 2013; Wood et al., 2015), confirming the tendency observed by authors who affirm that sexting behaviors are part of the rituals of adolescent courtship to flirt and engage in sexual-affective relationships (Lippman and Campbell, 2014). At the opposite end are striking percentages of adolescents who claim to send the images to a stranger (4.8%), the forwarding of images of a third party to these strangers (4.9%) and the reception of this type of images (12.2%) (Harris et al., 2013) which would confirm that the dissemination of this type of content is not exclusively done in an affective relationship but also among complete strangers.

Taking into account the gender of the participants, it has been found that sending images of intimate and sexual content is more likely to be produced by women (Mitchell et al., 2012) while the reception is more likely to occur among men (Hinduja and Patchin, 2010; Klettke et al., 2014). In this line, the results of the metanalysis show that women report being asked to send a sext more than men (Handschuh et al., 2019).

The absence of a unitary definition regarding sexting has involved the use of multiple instruments to estimate its occurrence in adults or adolescents:

(a) Sexting Behavior Scale (Dir, 2012): it is considered as a reference instrument by various studies and research whose objective is the measurement of sexting behavior. The scale measures the frequency and prevalence related to sending and receiving text messages or images of provocative or sexual content (sext) through mobile phones and social networks. Both the internal consistency (α = 0.883) and the reliability (α = 0.893) of the original scale are good, and the results are even improved in the Spanish adaptation of the scale (α = 0.923). As limitations, it can be pointed out the sample used to adapt it into the Spanish context, since a group of 661 adults between 18 and 51 years old was used to obtain their reliability results. Moreover, it mixes items with a different response format that need to be coded to analyze the results.
(b) Behavior on Sexting Scale (ECS) (Chacón-López et al., 2016): Based on the instrument proposed by Dir (2012) the authors propose a scale of 29 items to be scored by the subjects in a Likert scale of five points that oscillates from 0 (“never”/“not true”/“no exchange”) to 4 (“frequently”/“totally true”). The results obtained from a pilot study of 110 participants, and later by a larger sample composed of 985 subjects between 18 and 24 years old, indicate a very good internal consistency, both in the pilot test (α = 0.923) and in the final study (α = 0.922). The scale estimates the disposition, participation and emotional expression of sexting in young adults who have come of age, and for this reason the results of their psychometric properties and frequencies cannot be extrapolated to the adolescent population.
(c) Questionnaire adapted by Fajardo et al. (2013): Using as a base the adaptation of the questionnaire “Sex and Tech” carried out by the North American non-governmental agency (Power to Decide: The National Campaign to Prevent Teen and Unplanned Pregnancy, 2008) the authors use the Spanish adaptation (Questionnaire on Technology and Sexuality) (Marrufo, 2012), adding their own questions until completing a questionnaire with 45 items that assess the opinions and concerns of adolescent students about the use of mobile phones or the internet for sending or receiving provocative or suggestive messages, photos or videos on a Likert scale with four response options. The small size of the sample (132 adolescents between the ages of 13 and 17 belonging to schools in the town of Badajoz) means that the moderate reliability of the instrument (α = 0.752) in the Spanish population should be taken with caution until it is used in a more numerous and heterogeneous sample.
(d) Sexual risk behaviors and motivation toward online sex scales, (Vizzuetth et al., 2015): This instrument focuses not only on audiovisual content but focuses on sexting, sending messages with sexual or provocative content. It highlights a subscale within the test composed of items that intend to evaluate the reason why sexting behavior is performed in formal romantic relationships. The reliability obtained by means of a sample of 263 participants between 16 and 26 years old is very good, both for the scale of sexual risk behaviors (α = 0.962) and for the motivation scale (α = 0.909). The main limitation is related to the age of the participants, since adolescents under 16 years of age are out of this study and therefore there is no data available to assess, at a later stage, the need or not to carry out preventive measures regarding the conduct of sexting in the group of adolescents. Similarly, the description of the sexting motivation scale limits this evaluation to sexting behaviors with the formal partner, excluding behaviors of this type that can be performed with another subject (an acquaintance or a stranger).
(e) Sexting Attitudes Scale (Weisskirch and Delevi, 2011) formed by 17 items with a Likert type response format ranging from 1 (not at all true) to 5 (frequently true) and three factors (Fun and Carefree, Perceived Risk and Relational Expectations) with Cronbach’s Alpha coefficients ranging from 0.78 to 0.89. The adaptation of the scale into Spanish (Rodríguez-Castro et al., 2017) maintains the reliability results of the original scale with a reliability ranging between 0.77 and 0.83. Despite being an instrument adapted to the Spanish adolescent population, its objective is the measurement of attitudes toward sexting or its conceptualization, and it is not possible to infer the frequency or prevalence of such behaviors from its application.

Sending messages of intimate content, apart from the fact that it also occurs among the adult population, may also be consented and it may form part of the expression of the intimacy of an underage couple, which may have legal consequences depending on the age of the sender or receiver (Sacco et al., 2010; Wolak et al., 2012). Thus, in the recent meta-analysis of Madigan et al. (2018) and Handschuh et al. (2019) it has become clear that sexting is a phenomenon that has increased in recent years in the adolescent population. For all this, the realization of this type of behavior among minors is especially problematic.

Based on the previous results, the objectives of this study are the following:

(1) To develop a scale to estimate the frequency of the exchange of intimate and personal images among adolescents including aspects such as the sender, the receiver or its resubmission and request.
(2) To include in the previous scale not only people with whom the adolescents maintain some type of bond (friends or partners) but also people who are strangers to them.
(3) To include in the scale items referring to the publication of images of intimate and personal content on social networks.

## Materials and Methods

### Procedure and Participants

The procedure to collect information was carried out in Secondary Education Institutes of the Autonomous Community of Castilla y León (Spain) with which the researchers maintain institutional relations. The initial contact has been maintained with the school’s management. First, the objectives of the research were explained and a copy of the questionnaire to be used was provided. Once the endorsement of the management teams had been reached and after contacting with the teachers in charge of the different groups, the day of completion of the questionnaire by the adolescents was agreed upon.

To guarantee the representativeness of the sample, one age group was selected at random for each of the participating institutes (five in total), so that each of them had representation in all the age groups that were part of the final sample. On the day of the intervention, the students were gathered in the multimedia room of the different schools where each of the participants had access to an individual computer with an internet connection. In charge of a member of the research, a brief introduction has been made about the risks of social networks by minors and the different risk phenomena that can lead to their inappropriate use (grooming, sexting, etc.). Then it was explained that his participation was required for the completion of a questionnaire referred to the “Exchange of images of personal and intimate content through mobile devices and social networks” being this the definition of sexting used by researchers. It has been insisted that they should answer according to the general concept of social networks, being able to make the exchange and publication of images among others not indicated in the questionnaire and that were mentioned during the talk start (twitter, Pinterest, etc.). Similarly clarifications have been made to indicate that it was understood that instant messaging services (WhatsApp or Telegram among others) are considered within the section of exchange of images through mobile devices because of the need to have an associated telephone number. Likewise, the categories established for each of the groups formed have been clarified, understanding that strangers are those persons that the participants do not know or identify within their group of acquaintances and from whom the data through which they receive the request of images (phone number or profile on the social network) does not let them know if it is a person from their environment.

Once the clarifications have been completed, the questionnaire has been completed individually and through an online link, which has allowed the answers to be stored anonymously and without being able to identify the subject that issued them. The presence of a member of the research in each of the interventions had meant that only five questionnaires were discarded due to lack of response. The total application of the instrument did not exceed 30 min.

The sample consisted of students from the 1st to 4th levels of secondary education and from the two levels of high school education in Spain (equivalent to the 7th–12th grades and High School in the United States system) of each evaluated school. Parents of all participants were informed and offered the option to refuse to allow their children to participate in the study. Adolescents completed questionnaires during their regular class schedule. Participants were encouraged to ask questions that might arise when answering any of the items. Participation was voluntary and responses were anonymous to promote openness and honesty. The study was approved by the Ethics Committee of the University of A Coruña and the recommendations of the Helsinki Declaration and the General Data Protection Regulation (2016/679) approved by the European Parliament and the Council of the European Union have been followed. Written informed consent was obtained from all participants above the age of 16 and from the parents/legal guardians of all participants below the age of 16.

The final sample was formed by 602 adolescents aged between 12 and 19 years (*M* = 14.92, *SD* = 1.591), of which 282 (47.2%) are boys and 316 (52.8%) are girls and, in four cases, no information regarding gender has been obtained (see Table 1).

**Table 1:** Percentage of students by gender and academic year (*N* = 602).

Academic year	Total sample	Boys	Girls
7th Grade	13	46.8	53.2
8th Grade	11.8	42.9	57.1
9th Grade	46.9	54.9	45.1
10th Grade	24.9	48.6	51.4
11th Grade	14	45.2	54.8
12th Grade	9.5	29.8	70.2
Total		47.2	52.8

At a descriptive level, the results indicate that a large majority of the students surveyed have their own mobile phone (98.3%) compared to a minority that claims not to have it (1.7%), and where the youngest adolescents (7th and 8th Grade) are the ones that do not have their own mobile phone in a greater percentage. The data related to the privacy of the profile that adolescents have on social networks (public or private profiles) show that a percentage higher than 10% of participants claim to keep their profile open on social networks without any type of restriction, making the content or images published available on the Internet (see Table 2).

**Table 2:** Percentage of adolescents with mobile phone and the privacy of their profiles in social networks taking into account their gender and academic year (*N* = 602).

		They have their own mobile phone	They do not have their own mobile phone	Public profile	Private profile
Gender	Boys	98.2	1.8	12.7	87.3
	Girls	98.7	1.3	19.2	80.8
Academic year	7th Grade	93.6	6.4	14.5	85.5
	8th Grade	95.8	4.2	19.7	80.3
	9th Grade	99.4	0.6	13.5	86.5
	10th Grade	100	0	19.5	80.5
	11th Grade	98.8	1.2	14.5	85.5
	12th Grade	100	0	19.6	80.4

There are significant associations between gender and the type of profile they have in social networks, with a higher percentage of girls who do not have any type of restriction of access to their contents and images in these social networks (χ^2^(1,602) = 517.216, *p* < 0.001).

### Measures

To elaborate the Intimate Images Diffusion Scale between adolescents (Escala de Difusión de Imágenes Íntimas entre Adolescentes, EDIMA) 22 affirmations have been elaborated divided into two big factors. The factors have been established based on the nature of the interaction with the contents: instant messaging services through mobile devices or through digital platforms such as social networks.

Thus, the first factor includes 16 statements related to the reception, sending, requesting and re-sending of images and videos of intimate and personal content to partners, strangers, friends and, in general, without identifying the person, made through messaging services or chats installed on mobile phones. In this phase of operationalization of the variables, we have chosen to use the criterion of starting from the general to the particular, by including four initial items of a general nature, to help better discriminate the answers and to allow an approach to the subject less invasive for the respondent, as advised by experts in instrument construction (Dawis, 1987; DeVellis, 2016; Clark and Watson, 2019). Then, 12 items are presented, referring to the three types of relationship considered: partners, strangers, and friends.

The second factor consists of six statements. The first four focus on the frequency with which subjects have published images and videos of personal and intimate content through social networks (Instagram or Facebook, among others) of their partners, a stranger, friend or acquaintance or their own. The last two items refer to the frequency with which adolescents have received comments or humiliating messages in photos they have published, or if they have seen humiliating comments in photos of other people (see Appendix I).

### Statistical Analyses

The internal consistency of the scale was measured through the SPSS data analysis program (version 22 for Windows), based on the Cronbach alpha coefficient and Omega reliability coefficient.

In relation to the analysis of the factorial structure of the scale, the Confirmatory Factor Analysis (CFA) technique was applied. The use of Exploratory Factor Analysis (EFA) was dismissed since some authors recommend avoiding the use of this type of dimension reduction models when it comes to analyze scales that have been designed based on constructs or theoretical hypotheses that estimate a factorial structure beforehand (Ferrando and Anguiano-Carrasco, 2010). On the other hand, the CFA will allow testing the adjustment of the factorial model with regard to the theoretical model proposed, since it is about to contrast the convergence of the items analyzed in the factors proposed at the theoretical level in the process of the elaboration of the scale (Herrero, 2010). Thus, in the present study, the hypothesis of a factorial model of two factors was tested by applying a CFA to determine the goodness of fit of the factorial structure of the instrument through the model of structural equations with the statistical program AMOS (SPSS, version 22 for Windows).

To estimation the concurrent validity, the correlation with the Sexting Behavior Scale was analyzed. Finally, the prevalence of the behaviors collected in the items that make up the Intimate Images Diffusion Scale between adolescents (EDIMA) was estimated.

## Results

### Reliability Analysis

Reliability analyses of the whole scale, composed of 22 elements, showed optimal results (α = 0.969) (Supplementary Material). However, the analyses warned that greater reliability could be obtained with the elimination of two of the items that form the initial proposal. Apart from the statistical criteria, from the theoretical point of view, its elimination is recommended since it was observed that its content was not directly related to the dissemination of intimate images but to the comments that those images provoke (*I have received humiliating comments or messages on provocative photos, I’ve posted* and *I’ve seen humiliating comments on provocative photos of other people*). These items were eliminated according to the statistical and the theoretical criteria, obtaining an improvement of the analysis of reliability with the 20 items that form the final solution (α = 0.976), and the elimination of any of the remaining items does not mean an improvement of the analysis (see Table 3).

**Table 3:** Psychometric properties of the Intimate Images Diffusion Scale (EDIMA).

Item	*M-i*	*SD-i*	$r_{i}^{c}$	α*-i*	*M-i*	*SD-i*	$r_{i}^{c}$	α*-i*
1	41.20	143.401	0.645	0.970	36.37	116.012	0.639	0.978
2	41.75	144.731	0.812	0.968	36.93	117.002	0.819	0.975
3	41.69	144.797	0.775	0.968	36.87	117.122	0.778	0.975
4	41.77	144.534	0.862	0.967	36.95	116.793	0.875	0.974
5	41.64	143.390	0.749	0.968	36.82	115.849	0.751	0.975
6	41.74	143.765	0.813	0.967	36.92	116.132	0.819	0.975
7	41.87	146.502	0.885	0.967	37.05	118.581	0.897	0.974
8	41.83	145.348	0.864	0.967	37.01	117.491	0.874	0.974
9	41.54	143.802	0.719	0.969	36.72	116.393	0.712	0.976
10	41.87	146.075	0.896	0.967	37.05	118.204	0.907	0.974
11	41.78	145.311	0.813	0.968	36.97	117.537	0.822	0.975
12	41.86	145.762	0.889	0.967	37.04	117.933	0.896	0.974
13	41.62	143.123	0.799	0.968	36.80	115.833	0.789	0.975
14	41.80	144.462	0.865	0.967	36.98	116.817	0.870	0.974
15	41.83	145.584	0.844	0.967	37.01	117.796	0.852	0.974
16	41.85	145.537	0.906	0.967	37.03	117.786	0.912	0.974
17	41.78	145.538	0.785	0.968	36.96	117.966	0.775	0.975
18	41.90	147.181	0.883	0.967	37.08	119.291	0.886	0.974
19	41.89	146.919	0.841	0.968	37.07	119.021	0.847	0.974
20	41.88	147.178	0.831	0.968	37.06	119.354	0.828	0.975
21	41.80	144.798	0.809	0.974				
22	40.73	144.620	0.388	0.977				

*Item Total-Correlation (*$r_{i}^{c}$*), Cronbach’s alpha if item deleted (α-i), Mean if item deleted (M-i), Standard Deviation if item deleted (SD-i).*

Since the items of the scale are ordinal type and to obtain a more accurate measurement of the reliability of the instrument, the Omega reliability coefficient has been calculated through the free access computer program “R” version 3.1.2 (R Development Core Team, 2007), confirming the good results obtained (Ω = 0.981) and far exceeding the acceptable values established by other authors (Campo-Arias and Oviedo, 2008).

### Confirmatory Factor Analysis

In order to analyze the classification of the items that form the scale taking into account to the medium through which the dissemination, publication and sending of images of intimate content has been produced (mobile phone or social networks) has been carried out a confirmatory factor analysis with AMOS 22. The estimation method was Unweighted least squares (ULS) and in order to value the adjustment of the model, the following indices were used: goodness of fit index (GFI), adjusted goodness of fit index (AGFI), the root mean square residual index (RMR), the normed fit index (NFI), and the relative fit index (RFI). The estimation method used (Unweighted Least Squares) allows the variables do not follow a normal distribution and is especially recommended when the variables are of ordinal type (Morata-Ramírez et al., 2015), this being the reason why data on the normality of the sample are not included and the chi-squared index is not obtained.

The results show a good fit of the model composed by the factors that could be referred to as diffusion of intimate images through mobile devices and diffusion of intimate images through social networks (see Table 4). In accordance with authors such as Kline (2016) the values show a good model fit since RMR ≤ 0.06, and GFI, AGFI, NFI, and RFI > 0.90.

**Table 4:** Values of fit index of the model of the scale.

Statistical	Result
RMR	0.028
GFI	0.993
AGFI	0.991
NFI	0.992
RFI	0.991

**FIGURE 1:**
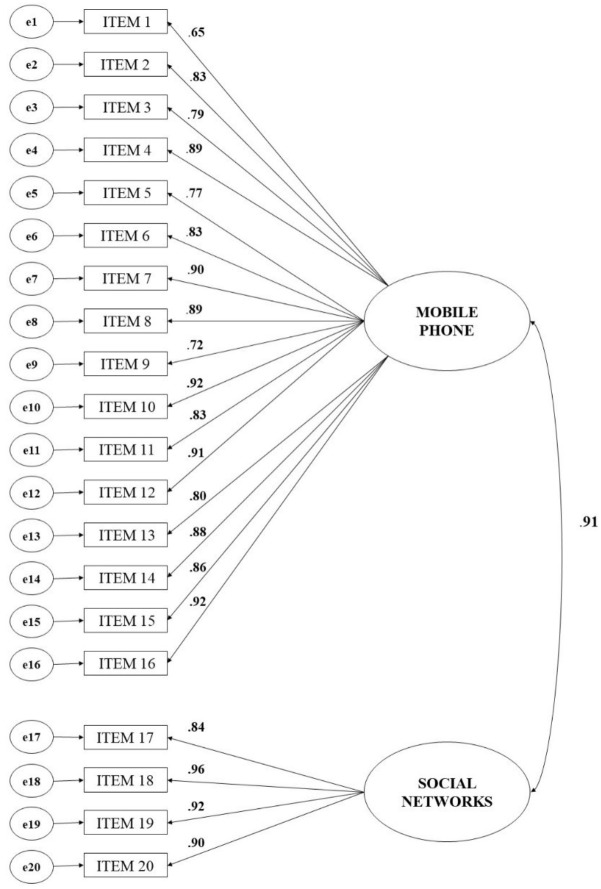
Confirmatory factor analysis of the Intimate Images Diffusion Scale (EDIMA).

All the items that form the factors significantly contribute to the explanation of it with factorial weights that range from 0.65 (I have received suggestive and provocative images/videos via mobile) to 0.96 (I have posted suggestive or provocative images/videos of my partner without their consent in social networks) (see Figure 1).

The theoretical model proposed to be tested by confirmatory factor analysis proves the existence of two factors or subscales that depend on the medium used by adolescents to diffuse or send images of intimate content: either through the mobile (16 items) or through their publication in social networks (4 items).

### Concurrent Validity

In order to analyze the validity of the elaborated scale, we have chosen to use the eight items of the Sexting Behavior Scale (Dir, 2012) that measure the frequency and prevalence related to the sending and receiving of sext (text messages or images of provocative or sexual content) via mobile phone and social networks, on a scale of five points ranging from 1 (“never”) to 5 (“frequently/every day”).

The correlation obtained between the Sexting Behavior Scale and the Intimate Images Diffusion Scale between adolescents (EDIMA), measured through the Pearson statistic, shows significant results (*r* = 0.729; *p* < 0.01). This indicates that the prevalence of both phenomena is related.

If a distinction by factors is made, the highest correlations are observed between the Sexting Behavior Scale and the factor of sending images of intimate content via mobile (*r* = 0.864; *p* < 0.01), while the correlation between the Sexting Behavior Scale and the factor of publication of images of intimate content via social networks, although significant, is smaller (*r* = 0.434; *p* < 0.01).

### Prevalence Data

The prevalence data obtained in each of the items that form the scale exceed 75% in all the behaviors, which means that the adolescents who have never participated in any behavior of dissemination or sending of images of intimate content are between 10 and 25%. The highest prevalence is found in behaviors considered as “passive” sexting that are related to the reception of images in general (89.5%), reception of provocative images of my partner (80.9%), reception of provocative images of a stranger (82.6%) and receiving provocative images from a friend and/or an acquaintance (81.2%). The active sexting (sending images and the forwarding and the request of these) maintain similar percentages of prevalence and, although to a lesser extent than in the passive sexting, they almost reach 80% of the participants.

Finally, the publication of images of intimate or personal content both of their own or of a third party with which some type of relationship (acquaintances or partners) or without any type of relationship is maintained, also reaches figures close to 80%, which indicates that these behaviors are commonly present among the adolescent population (see Table 5).

**Table 5:** Percentage of behaviors in each of the items (*N* = 602).

Item	Never	Rarely	Occasionally	Often	Frequently
1	10.6	45.7	30	9.7	4
2	20.6	67.7	8.2	1.3	1.8
3	19.4	19.6	10.1	2.5	1.7
4	20.8	69.3	7.2	1.2	1.5
5	19.4	65.2	9.3	3	3.2
6	21.3	67.6	6.9	1.7	9.5
7	22.9	73.7	1.7	0.7	1
8	22.7	70.9	3.9	1.2	1.3
9	17.6	59.3	15.1	5.7	2.3
10	23.2	73	2	0.7	1.2
11	21.3	71	4	2.2	1.5
12	23.1	72.5	2.2	0.8	1.4
13	19	61.8	12.9	4.4	1.8
14	22.7	68.4	5.9	1.3	1.7
15	22.2	72.3	3	0.8	1.7
16	22.3	72.7	2.8	0.8	1.3
17	22.7	67.7	6.8	1.7	1.2
18	23.7	74.8	0.3	0.3	0.8
19	24	74	0.5	0.3	1.2
20	23	74.3	1.2	0.5	1

Based on gender, it is observed that boys are the ones who most frequently perform behaviors of resending insinuating or provocative images/videos (item 3) [*t*(431.237) = 3.1315, *p* < 0.05], requesting insinuating or provocative images/videos (item 4) [*t*(427.389) = 2.607, *p* < 0.05], and the request of provocative images/videos to a friend (item 16) [*t*(420.720) = 2.104, *p* < 0.05]. It is girls who, more often than their male counterparts, post-suggestive or provocative images/videos on social networks (item 17) [*t*(554.998) = −2.137, *p* < 0.05] (see Table 6).

**Table 6:** Mean and standard deviations in each of the items based on gender.

	Boys		Girls	
	
Item	*M*	*SD*	*M*	*SD*
1	2.54	1.07	2.47	0.81
2	1.95	0.81	1.95	0.59
3	2.10	0.90	1.91	0.52
4	2.01	0.83	1.86	0.47
5	2.07	0.92	2.03	0.74
6	1.93	0.80	1.99	0.70
7	1.84	0.70	1.81	0.40
8	1.93	0.79	1.82	0.45
9	2.10	0.91	2.20	0.80
10	1.86	0.73	1.81	0.42
11	1.96	0.79	1.87	0.55
12	1.89	0.77	1.80	0.41
13	2.05	0.88	2.11	0.72
14	1.91	0.80	1.90	0.57
15	1.89	0.78	1.85	0.48
16	1.91	0.76	1.81	0.42
17	1.84	0.70	1.96	0.63
18	1.78	0.64	1.81	0.40
19	1.80	0.69	1.81	0.41
20	1.82	0.68	1.82	0.42

Finally, if we only take into account whether or not the subjects have made the exchange of images at some point (without taking into account the frequency of behavior), it is observed that the prevalence is greater among girls than boys (see Table 7).

**Table 7:** Percentage of occurrence of the summary variables according to gender (*N* = 602).

	Boys		Girls		
	
	Never	Ever	Never	Ever	*X*^2^
General	14.5	85.5	5.1	94.9	385.953^∗∗^
Partner	21.3	78.7	16.4	83.6	229.453^∗∗^
Stranger	19.7	80.3	13.6	86.4	263.116^∗∗^
Friend	21.4	78.6	14.9	85.1	240.435^∗∗^
Post	26.6	73.4	17.4	82.6	187.284^∗∗^

*^∗∗^p < 0.001.*

## Discussion

The aim of the present investigation was the elaboration of a scale that estimates the frequency with which adolescents exchange images of intimate and personal content through the mobile telephone or publish them on social networks. The results obtained indicated that the Intimate Images Diffusion Scale between adolescents (EDIMA) fulfills the objectives for which it has been created through the 20 items that compose it.

The advantage of the scale designed in this way was that it includes not only behaviors that are performed through the mobile phone or messaging services but also their publication on social networks. In the same vein, the scale distinguished if those images are of the person who publishes them, of people with whom a sentimental relationship is maintained, acquaintances or a complete stranger, to evaluate all the behaviors that can be performed with those images (sending, receiving, requesting, or re-sending).

The psychometric results of the Intimate Images Diffusion Scale between adolescents (EDIMA) showed the highest reliability of all the instruments designed so far to estimate the sending and dissemination of images of intimate or private content among Spanish adolescents. In addition to the psychometric properties, the elaborated instrument focused on the estimation of the frequency of objective behaviors of image exchange, differentiating it from questionnaires that focus on the motivation toward sexting behaviors (Vizzuetth et al., 2015) or in attitudes toward it or its conceptualization (Weisskirch and Delevi, 2011). The values obtained in the calculated goodness of fit indexes, in line with Hu and Bentler (1999) and Kline (2016), allowed us to conclude that the model shows a good fit.

The results obtained indicated that there is a high number of adolescents who, at some point, have performed the behavior of sending and receiving images or videos of provocative content, overcoming the prevalence observed so far for the sending of sexual messages, and indicating that this type of behavior is more frequent than indicated in other studies about sexting (Döring, 2014; Cooper et al., 2016; Gámez-Guadix et al., 2017). Similarly, the prevalence results obtained regarding the publication of images of intimate content through social networks that do not take into account a specific recipient indicated that this type of behavior, as well as the exchange of images through telephones mobile, was more frequent among the adolescents studied than the traditional forms of sexting.

The negative consequences of sexting that indicate a greater predisposition to show symptoms of depression, impulsivity and substance abuse (Temple et al., 2012, 2014), as well as a lower self-esteem among adolescents who perform this type of behavior (Ybarra and Mitchell, 2016), and the high concurrence between this phenomenon and the diffusion of images of intimate content through social networks and mobile devices, means that an increasing percentage of adolescents suffering from the aforementioned disorders can be assumed. Sexting has been also relating to a high risk of dating violence (Morelli et al., 2016) and sextortion (Hinduja and Patchin, 2018) and due to its psychosocial and legal consequences, Van Ouytsel et al. (2019) consider sexting as a public health issue. Bearing this in mind, EDIMA can be useful to detect and estimate sexting among adolescents on clinical, forensic and educational practice. Youth-serving professionals plays an important preventive role (Hinduja and Patchin, 2018) and EDIMA scale can help them to detect sexting and estimate the need of educational interventions about social media use or sexual behavior. Thus, the design of the Intimate Images Diffusion Scale between adolescents (EDIMA), with response format based on the frequency of the type of behavior, also allow the early establishing of risk groups that make it possible to perform an intervention based on the severity of the adolescents’ behavior.

Taking into account the gender, a greater percentage of women who at least have sent, received, resent, requested or published images of intimate content in comparison with the boys is observed, and this result was in line with what was noted by previous studies (Mitchell et al., 2012). Considering the frequency with which the behaviors described in the scale occur, it can be concluded that the reception of images of intimate and sexual content is more likely to occur among men (Hinduja and Patchin, 2010; Klettke et al., 2014), these results can be explained from the fact that they are the ones who, with a higher frequency than the girls, resend and request for images of this type and, therefore, they are also more likely to receive them. A possible explanatory hypothesis can derive from the socially accepted behaviors for each gender role and where there is a series of socially learned behaviors, in which boys, in matters of a sexual nature, continue to have a role of domination and girls of submission; and were girls are more predisposed than boys to expose themselves, attending to canons of beauty and success linked to the image.

Based on the above, it can be concluded that the elaborated scale fulfilled the objective of being a reliable instrument for the estimation of an increasingly frequent phenomenon in adolescents, the publication and exchange of images of intimate and personal content, becoming an independent construct differentiated from the exchange of messages of sexual content (sexting). Future research must aboard the impactions of sharing intimate and personal contents in the adolescent’s socialization process and its consequences on their wellbeing.

## Data Availability

The raw data supporting the conclusions of this manuscript will be made available by the authors, without undue reservation, to any qualified researcher.

## Ethics Statement

The study was approved by the Ethics Committee of the University of A Coruña and the recommendations of the Helsinki Declaration and the General Data Protection Regulation (2016/679) approved by the European Parliament and the Council of the European Union have been followed. Written informed consent was obtained from all participants above the age of 16 and from the parents/legal guardians of all participants below the age of 16.

## Author Contributions

All authors listed have made a substantial, direct and intellectual contribution to the work, and approved it for publication.

## Conflict of Interest Statement

The authors declare that the research was conducted in the absence of any commercial or financial relationships that could be construed as a potential conflict of interest.

## Appendix I

**Appendix I:** Intimate Images Diffusion Scale between adolescents (Escala de Difusión de Imágenes Íntimas entre Adolescentes, EDIMA).

A series of behaviors that can be performed via **social networks** and that have to do with the **diffusion of images of intimate and/or personal content** are presented here below. Please indicate in each of the items that is presented **the frequency with which you have performed each of the behaviors** according to the following scale:					

**1. Never** **2. Rarely** (once or twice a month) **3. Occasionally** (2 or 3 times a month) **4. Often** (2 or 3 times a week) **5. Frequently** (Every day)					
The data collected are totally **CONFIDENTIAL** and neither the teachers nor your parents will have access to them, so we ask you to be **TOTALLY SINCERE** inyour answers.					

Via **mobile phone** (chats)					
(1) I have **received** *suggestive or provocative images/videos*	1	2	3	4	5
(2) I have **sent** *suggestive or provocative images/videos* of my own	1	2	3	4	5
(3) I have **resent** *suggestive or provocative images/videos*	1	2	3	4	5
(4) I have **requested** *suggestive or provocative images/videos*	1	2	3	4	5
(5) I have **received** *intimate or provocative images/videos* of my **partner**	1	2	3	4	5
(6) I have **sent** *intimate or provocative images/videos of my own* to my **partner**	1	2	3	4	5
(7) I have **resent** *intimate images/ videos of* ***my partner*** *without their consent* to a **third person**	1	2	3	4	5
(8) I have **requested** *suggestive or provocative images/videos* to my partner	1	2	3	4	5
(9) I have **received** *suggestive or provocative images/videos* from a **stranger**	1	2	3	4	5
(10) I have **sent** *suggestive or provocative images/videos* of my own to a **stranger**	1	2	3	4	5
(11) I **have resent** *intimate images/ videos* of a stranger *without their consent* to a third person	1	2	3	4	5
(12) I have **requested** *suggestive or provocative images/videos* to a stranger	1	2	3	4	5
(13) I have **received** *intimate or provocative images/videos* from a **friend** or an acquaintance	1	2	3	4	5
(14) I have **sent** *intimate or provocative images/videos* of my own to a **friend** or an acquaintance	1	2	3	4	5
(15) I have **resent** *intimate images/videos* of a friend or an acquaintance to a third person without their consent	1	2	3	4	5
(16) I have **requested** *suggestive or provocative images/videos* to a **friend** or an acquaintance	1	2	3	4	5
Via **social networks** (Instagram, Facebook)					
(17) I have posted suggestive or provocative images/videos of my own	1	2	3	4	5
(18) I have posted suggestive or provocative images/videos of my partner without their consent	1	2	3	4	5
(19) I have posted suggestive or provocative images/videos of a stranger without their consent	1	2	3	4	5
(20) I have posted suggestive or provocative images/videos of a friend or an acquaintance without their consent	1	2	3	4	5
